# Radiologic discrepancies in diagnosis of fractures in a Dutch teaching emergency department: a retrospective analysis

**DOI:** 10.1186/s13049-020-00727-8

**Published:** 2020-05-13

**Authors:** Laura Mattijssen-Horstink, Judith Joëlle Langeraar, Gert Jan Mauritz, William van der Stappen, Maarten Baggelaar, Edward Camillus Thwan Han Tan

**Affiliations:** 1 Department of Emergency Medicine, Radboudumc, Geert Grooteplein Zuid 10, 6525 GA Nijmegen, The Netherlands; 2 Department of Emergency Medicine, Rijnstate, Arnhem, The Netherlands; 3 Department of Surgery, Canisius-Wilhelmina Ziekenhuis, Nijmegen, the Netherlands; 4 Department of Emergency Medicine, Canisius-Wilhelmina Ziekenhuis, Nijmegen, The Netherlands; 5 Department of Surgery, Radboudumc, Nijmegen, The Netherlands; 6 Department of Emergency Medicine, Radboudumc, Nijmegen, The Netherlands

**Keywords:** Emergency department, Fracture, Radiographs, Radiologic discrepancies, Missed diagnosis, Diagnostic error

## Abstract

**Background:**

Missed fractures in the emergency department (ED) are common and may lead to patient morbidity.

**Aim:**

To determine the rate and nature of radiographic discrepancies between ED treating physicians, radiologists and trauma/orthopaedic surgeons and the clinical consequences of delayed diagnosis. A secondary outcome measurement is the timeframe in which most fractures were missed.

**Methods:**

A single-centre retrospective analysis of all missed fractures in a general teaching hospital from 2012 to 2017 was performed. Data regarding missed fractures were provided by the hospital’s complication list and related database. Additional data were retrieved from the electronic medical records as required for the study.

**Results:**

A total of 25,957 fractures were treated at our ED. Initially, 289 fractures were missed by ED treating physicians (1.1%). The most frequently missed fractures were the elbow (28.6%) and wrist (20.8%) in children, the foot (17.2%) in adults and the pelvis and hip (37.3%) in elderly patients. Patients required surgery in 9.3% of missed fractures, received immobilization by a cast or brace in 45.7%, had no treatment alterations during the first week in 38.1%. Follow-up data were lacking for 6.9% of cases. 49% of all missed fractures took place between 4 PM and 9 PM. There is a discrepancy in percentages of correctly diagnosed fractures and missed fractures between 5 PM and 3 AM.

**Conclusion:**

Adequate training of ED treating physicians in radiographic interpretation is essential in order to increase diagnostic accuracy. A daily multidisciplinary radiology meeting is very effective in detecting missed fractures.

## Introduction

Failure to diagnose a fracture accounts for up to 80% of emergence department (ED) diagnostic errors and is a leading cause of litigation [1, 2]. Radiography remains the initial modality used to detect a fracture. In the Netherlands, most EDs in general hospitals do not have a radiologist available 24/7. Therefore, radiographs are initially interpreted by ED treating physicians, and clinical practice is dependent on these interpretations. A recent study by Gergenti et al., performed during ‘out of office’ hours, showed that missed fractures are among the most common radiology error, even for radiology residents and staff [3]. In the literature, figures can be found regarding radiologic errors among radiologists and ED treating physicians (1.4–2.4%), Department of Surgery interns (3.1%), orthopaedists (2.5%) and radiology residents (1.8%) [4–9]. Existing literature illuminates little regarding the radiologic error rates for ED treating physicians, radiologists and trauma/orthopaedic surgeons in fracture evaluation in the Netherlands.

Therefore, this study aims to determine the rate of error for ED treating physicians, radiologists and trauma/orthopaedic surgeons in the interpretation of performed radiographs for possible fractures. Additionally, we examine the timeframe in which most fractures were missed. Finally, we want to identify the most common diagnostic errors and investigate the clinical consequence of delayed diagnosis.

## Method

The Canisius-Wilhelmina Hospital in Nijmegen, a city of 170,000 habitants in the Netherlands, is a level 2 teaching hospital with a tertiary level 1 trauma centre close by. An out-of-hours primary care centre is located next to the emergency department (ED). The ED receives approximately 25,000 patients a year. Since 2008, our ED is staffed with emergency physicians who supervise junior doctors, and since 2011, there has been 24/7 coverage by emergency physicians.

For this retrospective case series, fractures missed by ED treating physicians interpreting radiographs from January 2012 to December 2017 were included. We also performed a sub-analysis of missed fracture diagnoses when no radiograph was performed during the initial ED visit.

Radiographs are viewed by ED treating physicians using a Picture Archiving and Communication System on a high-resolution monitor. Fractures are marked by an arrow and these plain films are saved for reading by the radiologist. When the radiologist spots a fracture that is not marked by ED treating physicians, they call the emergency physician. Radiologists are available at the hospital Monday–Friday during daytime hours, and an on-call radiologist is available after 5 PM and during weekends.

A missed fracture was defined as a radiograph that is read as normal by ED treating physicians but as showing a fracture by either a radiologist or a trauma/orthopaedic surgeon. Additionally, separate unidentified fractures, apart from the initial fracture, were scored as missed fractures. When a patient had multiple fractures that were missed, each fracture was counted separately.

Since January 2012, complications have been registered using a complication form, collected by one of the emergency physicians and processed in a database. Under this system, there are five possible ways for detecting missed fractures. The forms are either filled in during the ED radiology meeting, which is held every weekday morning and is staffed by at least one emergency physician and a trauma or orthopaedic surgeon. The radiographs performed Friday–Sunday are reviewed on Monday. Another route is by phone call from a radiologist who reports the radiograph or CT-scan and detects a missed fracture. Radiographic reading by radiologists takes place within 4 days of an ED visit. Approximately 1 week after the trauma occurs, most patients with trauma to the extremities go to an outpatient follow-up at the trauma outpatient clinic, which is staffed by the same trauma or orthopaedic surgeons who attend the radiology meetings. When a missed fracture is detected by a radiologist or orthopaedic surgeon, the complication form is sent to the ED. The final routes are self-referral to the ED or referral by a general practitioner – either to the ED or the trauma outpatient clinic. When the diagnosis changes, either the emergency physician or trauma/orthopaedic surgeon informs the patient.

Data regarding age, gender, time of arrival at the ED, missed fracture, timeframe of the missed fracture, diagnosis by radiologist and/or trauma/orthopaedic surgeon and the clinical relevance were retrieved from the electronic medical record.

For sub-analyses, the study sample was divided into age groups (0–14 years old, 15–64 years old and ≥ 65 years old), in order to facilitate analysis of paediatric fractures and fractures in patients with possible osteoporosis. For ease of literature comparison, we set the age group for paediatric fractures at 0–14 years. Data regarding correctly diagnosed fractures were retrieved using ICD-9 coding. We used the Chi square test to compare proportions of missed fractures in the study sample of 2012–2014 and 2015–2017.

All data were analysed using the Statistical Package for Social Sciences (SPSS, Inc., IL, USA), version 25.

## Results

From 1 January 2012 to 31 December 2017, a total of 25,957 fractures were treated at our emergency department (ED). Other minor trauma diagnoses in our study period, wounds excluded, stood at 12,432 contusions (25%), 8105 joint distortions (17%) and 2485 joint dislocations (5%). Overall, a fracture was present in 53% of patients with a trauma-related ED visit and a possibility of a fracture, but in females and males aged 65 and older, these figures were 72% (5022 out of 7020 patients) and 63% (1578 out of 2499 patients), respectively.

We collected 339 cases of missed fractures, 50 (14.7%) of which were missed diagnoses, for which no radiograph was performed during initial ED visit. In 24% of missed diagnoses, examination of the fractured site was initially not documented. In 52% of cases, there was minor or no tenderness documented at the site in which a fracture was discovered at revisit. Physical examination was complicated by alcohol intoxication, learning/cognitive disabilities or dementia in at least 14% of cases. The missed diagnosis was a second, third or fourth fracture in 38% of cases. At revisit, all missed fractures were recognised by treating physicians at performed radiographs. These 50 missed diagnoses were excluded from further analyses.

Thus, 289 radiographic fractures missed by ED treating physicians remain (1.1% of the total number of fractures). There were 77 missed fractures in children (0–14 years old). With a total of 6740 fractures in this age group, 1.1% of all paediatric fractures were missed. Table 1 shows the number of missed cases a year. Using the Chi square test to compare the proportions of missed fractures between 2012 and 2014 and 2015–2017, it shows a significant decrease in missed fractures (*p* < 0.0001). Our study population consisted of 277 patients, as multiple fractures were missed in some patients. The median age of this sample was 35 years (range 0–93). Of these patients, 49.1% were men.

**Table 1:** Missed fractures per year

Year	Fractures
2012	55/4236 (1.3%)
2013	69/4288 (1.6%)
2014	61/4405 (1.4%)
2015	32/4465 (0.7%)
2016	44/4452 (1%)
2017	28/4111 (0.7%)
Total	289/25957 (1.1%)

**Fig. 1:**
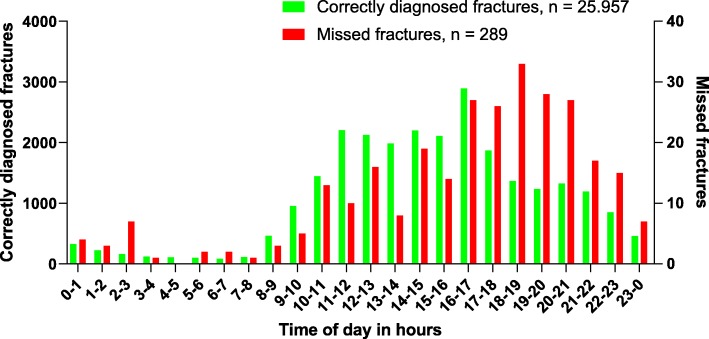
Total amount of correctly diagnosed and missed fractures per hour of a day

The greatest number of fractures were diagnosed between 10 AM and 10 PM. The amount of missed fractures, as well as the amount of correctly diagnosed fractures, over the course of the day is presented in Fig. 1. A striking 71.6% of missed fractures took place between 2 PM and 11 PM, with a peak between 4 PM and 9 PM (49% of all missed fractures). When we calculate the percentages of correctly diagnosed and missed fractures per timeframe, there is a discrepancy in these percentages between 5 PM and 3 AM.

There was a great diversity in radiographic missed fractures, with 22 different fractures missed only once or twice, for example, a sternal fracture, an avulsion fracture of the acromion and a fracture of the talus. Figures regarding missed fractures by anatomic location are presented in Table 2.

**Table 2:** Missed fractures by anatomic location, calculated per age group and as overall population

	0 to 14 years	15 to 64 years	65 years and older	Overall
Facial bones	–	1 (0.7%)	3 (4.5%)	4 (1.4%)
Shoulder	1 (1.3%)	3 (2.1%)	7 (10.4%)	11 (3.8%)
Elbow	22 (28.6%)	15 (10.3%)	–	37 (12.8%)
Wrist	16 (20.8%)	17 (11.7%)	5 (7.5%)	38 (13.1%)
Hand and fingers	12 (15.6%)	23 (15.9%)	3 (4.5%)	38 (13.1%)
Pelvis and hip	–	9 (6.2%)	25 (37.3%)	34 (11.8%)
Knee	4 (5.2%)	13 (9%)	2 (3%)	19 (6.6%)
Ankle	9 (11.7%)	20 (13.8%)	8 (11.9%)	37 (12.8%)
Foot	12 (15.6%)	25 (17.2%)	4 (6%)	41 (14.2%)
Thorax	–	7 (4.8%)	5 (7.5%)	12 (4.2%)
Spine	1 (1.3%)	12 (8.3%)	5 (7.5%)	18 (6.2%)
Total	77 (100%)	145 (100%)	67 (100%)	289 (100%)

Overall, 73% of missed fractures were diagnosed within 24 h of the ED visit. A large majority of these fractures were detected at the radiology meeting. The clinical relevance of the missed fractures is presented in Table 3.

**Table 3:** Patient consequences of missed fractures

Treatment started after detection of missed fracture	pediatric	adults	overall
Surgery	6 (7.8%)	21 (9.9%)	27 (9.3%)
Immobilization by cast or brace	38 (49.3%)	94 (44.3%)	132 (45.7%)
No changes apart from outpatient follow up	29 (37.7%)	81 (38.2%)	110 (38.1%)
Unknown	4 (5.2%)	16 (7.6%)	20 (6.9%)
	77 (100%)	212 (100%)	289 (100%)

Table 4 shows the missed fractures with indications for surgical treatment. One notable case is a patient who received a negative radiograph and negative CT-scan of the pelvis and hip for a clinically suspected upper femoral fracture and revisited the ED 2 weeks later with a subcapital femoral neck fracture. From the medical record, it is unclear whether there had been another trauma. At initial visit, no MRI scan was performed, as this is not common practice in the Netherlands.

**Table 4:** Surgical indication by type of fracture and timeframe from ED visit

Type of fracture	< 24 h of ED visit	24 h - 1 week of ED visit	1–6 weeks of ED visit
Greater tuberosity fracture			1
Pediatric supracondylar or transcondylar fracture of the humerus	4	1	
Distal radial Salter-Harris type II fracture	1		
Bennett fracture			1
Volar plate injury	1		
Acetabular fracture	1		
Subcapital femoral neck fracture	5	2	3
Intertrochanteric fracture	1		
Tibial plateau fracture	2		
Patellar tendon avulsion fracture	1		
Weber C or maisonneuve fracture	1	1	
Lisfranc injury	1		
	18/27 (67%)	4/27 (15%)	5/27 (18%)

From 2012 until 2017, a total of 38 acetabular fractures were seen at the ED. Seven of these fractures (18%) were initially missed. The majority of these patients were admitted to the hospital, and the fracture was discovered by CT scan the next morning. Calcaneal fractures were also frequently missed (15/105 fractures, 14%). In children, five supracondylar and eight medial epicondylar fractures of the humerus were missed. Most children had already received a cast as pain reduction when their fractures were discovered. For four patients, surgery on a displaced medial epicondylar fracture was performed within 24 h of ED visit. Ten out of 1450 collum femoris fractures were missed (0.7%). These 10 patients were aged 59 and older. In another three patients, no radiograph was performed during the initial ED visit, because these patients were able to walk. These three patients revisited the ED within 1 week of their initial ED visit.

The attending radiologist who reads the radiograph missed a fracture in 94/289 cases (32.5%). Of these missed fractures, 59 were detected by the trauma/orthopaedic surgeon at the radiology meeting or at the trauma outpatient clinic. Thus, 35/289 (12.1%) fractures were missed by ED treating physicians, attending radiologists and attending trauma/orthopaedic surgeons.

## Discussion

This large retrospective study shows that the rates of radiologic errors between our emergency department (ED) treating physicians, radiologists and trauma/orthopaedic surgeons are quite low. Missed fractures at the ED are common and range from 1.4–3.7% overall and 0.3–5.9% in paediatric populations [4, 6, 8, 10–12]. Therefore, our incidence of 1.1% for both paediatric and overall population is comparable to the incidence of other institutions. During the study period, missed fractures were presented to and discussed by emergency physicians multiple times. The overall decreasing trend in missed fractures that emerges throughout the study period is possibly due to a learning effect. In our study population, a fracture was more frequently present in elderly patients, especially in females, probably due to osteoporosis.

A study by Guly found 13.4% of missed fractures were due to failure to radiograph [1]. In our population, 14.7% of missed fractures were due to not performing a radiograph during initial ED visit. In the majority of these cases there was no clinical indication for radiography at initial visit. In the Netherlands, it is common to employ a ‘wait and see’ policy in clinical non-suspect fracture injuries, thus no radiographs were routinely made to exclude fractures. Rather, patients are advised to return to the trauma outpatient clinic in case of ongoing complaints.

Our main finding was a discrepancy between the percentages of correctly diagnosed and missed fractures between 5 PM and 3 AM, with a peak between 5 PM and 11 PM. In our study, the majority of patients with missed fractures visited the ED between 2 PM and 11 PM. We found one article by Hallas regarding missed fractures and time of radiography at a Norwegian ED. In this study, the incidence of missed fractures was highest (47%) between 8 PM and 2 AM [4]. The most likely explanation for the higher incidence of missed fractures after office hours is crowding at the ED during early evening hours. Another explanation could be a higher threshold for consulting the attending radiologist, as they are only available on call at those times. At our hospital, junior doctors are allowed to release patients from the ED without review of a radiograph by the supervising emergency physician, although many junior doctor do consult the emergency physician. A large study by Guly regarding diagnostic errors at the ED showed most fractures (85.3%) are missed by junior doctors [1]. Therefore, it is advisable that junior doctors request a second look by the emergency physician with a radiograph. Being aware of the timeframe for most frequently missed fractures may encourage emergency physicians to take a little more time for radiography reading in these hours.

Our third objective was to examine the most common diagnostic errors. According to available literature, there is some discrepancy regarding anatomical regions where fractures are especially likely to be missed. In some studies, no specific anatomical region is found to be significant [4], while others found the elbow (12.2–30%), hands and fingers (8.7–22%), and ankle and foot (17.9–35.1%) to be the anatomic districts in which fractures are most frequently missed [4–6, 8, 13]. In paediatric patients, the phalanges of the hand (22–26.4%) and the elbow (11.4–15.3%) are the sites of most frequent misdiagnosis [8, 14]. Our missed fractures occurred in accordance with these figures. Overall, the pelvis and hip region account for 11.8% of missed fractures, except in the age group 65 years and older – where they account for 37.3%. A substantial part of these missed fractures are femoral neck fractures. The incidence of radiographic occult hip fractures ranges from 2 to 10% in patients presenting with pain after trauma. An MRI or CT scan is advised in patients with a clinical suspicion of a non-displaced hip fracture and a negative radiograph [15–18]. Scanning could have reduced the delay in correctly diagnosing our patients and diminished patient discomfort.

Our final research question regards the clinical implication of delayed diagnosis. In 38.1% of cases, the fracture diagnosis did not lead to a change in treatment during the first week, mostly because the emergency physician already initialized the correct treatment. In the literature, in 46–86% of cases treatment was altered [4, 8]. Calculated for the whole population, discrepancy between radiographic reads led to treatment alterations in 1–3% of cases [1, 8]. In our study, 159/25,957 (0.6%) fractures led to altered treatment strategies. Follow up was unavailable in 20 missed fractures (7%). Assuming there should have been changes in treatment in these cases, our figure of 0.7% (179/25.957) treatment alteration in fractures is well below other institutions. A possible explanation could be the establishment of correct treatment by emergency physicians based on clinical examination. For instance, all patients with a clinical suspicion of a scaphoid fracture received a cast. In this study, we did not examine false positive fracture rates.

Missing a fracture can lead to major clinical impacts. In our study, 9.3% underwent surgical management. Divided by age group, paediatric missed fractures needed surgery in 7.8% of cases (6 out of 77 patients). This is in line with a study by Arora regarding paediatric patients, in which 3/25 (12%) missed fractures by emergency physicians and radiology residents required surgery [12]. In five of our paediatric patients, surgery was performed on a dislocated medial epicondylar fracture of the humerus. Medial epicondylar fractures accounted for approximately 10% of paediatric elbow fractures. In our study, no lateral condylar fractures were missed and supracondylar fractures were less frequently missed compared to medial epicondylar fractures. To reduce radiographic errors by ED treating physicians, a routinely obtained comparison radiograph of the asymptomatic elbow region could be performed in cases where doubt exists. Further, imaging by MRI or CT scan could also be necessary. The role of ultrasound of the elbow in the emergency setting has yet to be established [19, 20].

To detect missed fractures by ED treating physicians, the radiographic interpretation by the radiologist is usually considered the gold standard. Although it was not our intention to judge the performance of radiologists, it is striking that 32.5% of our missed fractures were also missed by the attending radiologist. Traditionally, it is expected that the radiologist has superior pattern recognition skills compared with ED treating physicians. Nevertheless, the effect of clinical history on radiological interpretation is massive, and we did not investigate the (frequently brief) history provided to the radiologist by the ED treating physicians [21]. Overall, 35 fractures (12.1%) were missed by ED treating physicians, radiologists and trauma/orthopaedic surgeons. In 12/35 cases (34%), the fracture was not visible, in retrospect, at the initial radiograph. In 6/35 cases (17%), the missed fracture was the second or third fracture to the patient, pointing out the importance of being aware of satisfaction of search.

There are several limitations to this study. First, we performed a single-centre retrospective analysis of missed fractures in a teaching hospital. Results may not be generalizable to other EDs in the Netherlands, as not all EDs are staffed with emergency physicians 24/7 and there is a great variability in the training level of junior doctors – from just graduated medical school to multiple years of working experience. Furthermore, due to the retrospective character of this study, it was impossible to assess the degree of education of the ED treating physician who initially has read the radiograph. Finally, there are several possibilities for selection bias. Although complication forms are present at the ED and the trauma outpatient clinic, selection bias is possible by not fulfilling all requirements of the forms. Furthermore, we probably underestimate the incidence of missed fractures because of the retrospective character of this study and the possibility of patients going to another hospital. The Netherlands is a densely populated country with multiple hospitals within a 1 hour drive of each other. If a fracture is missed by both emergency physician and radiologist or trauma/orthopaedic surgeon, detection of a missed fracture is only possible if the patient seeks further medical attention, which is unlikely to occur in minor injuries.

## Conclusion

This study shows the importance of an adequate safety net for ED treating physicians when reading radiographs. Adequate training of ED treating physicians is essential in order to increase diagnostic accuracy. Extra attention should be given to reading radiographs during afternoons and evenings, since most fractures were missed in these hours. Finally, we want to emphasise the importance of clinical information entered into the request form for the radiograph, since a lack of clinical information is most likely the cause of many of the fractures missed by our radiologists.

## Data Availability

The datasets used and/or analysed during the current study are available from the corresponding author on reasonable request.

## Abbreviations

ED: Emergency department
SPSS: Statistical Package for Social Sciences
SD: Standard deviation
AM: Ante Meridiem
PM: Post Meridiem
CT: Computed tomography
MRI: Magnetic resonance imaging
